# New Prospective for the Management of Low-Risk Pulmonary Embolism: Prognostic Assessment, Early Discharge, and Single-Drug Therapy with New Oral Anticoagulants

**DOI:** 10.6064/2012/502378

**Published:** 2012-12-17

**Authors:** Alessandro Squizzato

**Affiliations:** Research Center on Thromboembolic Disorders and Antithrombotic Therapies, Department of Clinical and Experimental Medicine, University of Insubria, Varese, Italy

## Abstract

Patients with pulmonary embolism (PE) can be stratified into two different prognostic categories, based on the presence or absence of shock or sustained arterial hypotension. Some patients with normotensive PE have a low risk of early mortality, defined as <1% at 30 days or during hospital stay. In this paper, we will discuss the new prospective for the optimal management of low-risk PE: prognostic assessment, early discharge, and single-drug therapy with new oral anticoagulants. Several parameters have been proposed and investigated to identify low-risk PE: clinical prediction rules, imaging tests, and laboratory markers of right ventricular dysfunction or injury. Moreover, outpatient management has been suggested for low-risk PE: it may lead to a decrease in unnecessary hospitalizations, acquired infections, death, and costs and to an improvement in health-related quality of life. Finally, the main characteristics of new oral anticoagulant drugs and the most recent published data on phase III trials on PE suggest that the single-drug therapy is a possible suitable option. Oral administration, predictable anticoagulant responses, and few drug-drug interactions of direct thrombin and factor Xa inhibitors may further simplify PE home therapy avoiding administration of low-molecular-weight heparin.

## 1. Introduction

Prognostic assessment is central in the initial management of patients with acute pulmonary embolism (PE) [1–3]. The latest guidelines of the European Society of Cardiology (ESC), and of the American College of Chest Physicians (ACCP), American Heart Association (AHA) scientific statement on PE strongly suggest to stratify PE patients into two categories, which are patients at high risk of early mortality, that is, massive PE, and patients who are not at high risk of early mortality, that is, submassive and low-risk PE, based on the presence or absence of shock or sustained arterial hypotension, respectively, [1–3]. Early prognostic stratification is therefore required to identify those patients who may be theoretically eligible for outpatient treatment or early discharge [4–6] and those patients who may require more aggressive therapeutic strategies [1–3].

Aim of this paper is to summarize current evidence on the best management of PE patients at low risk of adverse outcomes. In particular, available prognostic tools, home-treatment and early discharge, and new drug options will be discussed. 

## 2. Prognostic Assessment

Risk stratification of PE patients may assist clinicians in determining the best treatment and the appropriate setting for the initial therapy [3]. PE patients are commonly admitted to hospital for their initial treatment, though some of them may be suitable for a short-hospital stay or a complete home treatment [4, 5]. The availability of simple tools that accurately predict short-term adverse outcomes after the diagnosis of PE would be extremely valuable for the practicing clinicians. 

Patients with acute massive PE, that is, presenting with sustained hypotension or requiring inotropic support, pulselessness, or persistent profound bradycardia, have the highest risk of short-term mortality (>50%) [7]. These patients require hospital admission and administration of pharmacological thrombolysis or, in case of contraindication, cardiac surgery [1–3].

Patients not at high risk of early mortality are highly heterogeneous and are >90% of patients with PE [1]. Among them, a group can be identified with associated low risk of early mortality (defined as ~1%, at 30 day or inhospital), whereas the group at intermediate risk can have an early mortality rate up to about 15% [7]. Several parameters have been proposed and investigated for PE prognostic stratification: demographical, anamnestic, and objective findings (often combined in clinical prediction rules (CPRs)) and imaging tests and laboratory markers of right ventricular (RV) dysfunction or injury [1–3]. The AHA has defined low-risk PE as follows: acute PE and the absence of the clinical markers of adverse prognosis that define massive or submassive PE [2]. However, clinical markers show different prognostic accuracy to stratify PE patients.

### 2.1. Clinical Prediction Rules

CPRs are based on clinical data that are collected routinely, therefore being easy to obtain and widely applicable. Nine clinical CPRs were developed in recent years [8]. The pulmonary embolism severity index (PESI) and the easier version, the simplified PESI [9–26] and the Geneva prognostic CPR [27–31] are the most rigorously derived and validated CPRs (Tables 1 and 2). In particular, PESI takes into account age, gender, presence of cancer, heart failure, chronic lung disease, tachycardia, hypotension, tachypnea, low body temperature, altered mental status, and hypoxia. In particular, PESI identifies about 40% of PE patients (PESI class I and II) with an inhospital mortality of less than 1% (0.2%, 95% confidence interval (CI) 0–0.7%) [8], that is, both the ESC and the AHA threshold for defining PE patients at low-risk. 

### 2.2. Right Ventricular Dysfunction or Injury

Systematic reviews of the literature have shown that patients with right ventricular dysfunction on echocardiography, elevated levels of brain natriuretic peptide (BNP), NT-pro-BNP, or troponins had an increased risk of adverse inhospital outcomes as compared with patients with normal levels [32–34]. When used to identify low-risk patients, normal levels of BNP or NT-pro-BNP were associated with an early mortality rate of 2.2% (95% CI, 0.45–6.2) and 1.3% (95% CI, 0.15–4.4), respectively, with an overall rate of adverse outcomes of 13% (95% CI, 9.1%–19.0) and 5.3% (95% CI, 2.8–9.1) [32, 35–48]. In patients with normal troponin levels, early mortality rate was 3.7% (95% CI, 2.7–4.7), and the adverse outcome rate was 14.7% (95% CI, 10.8–18.6) [33, 48–54]. Echocardiographic signs of right ventricular dysfunction had a 60% negative predictive value (95% CI, 55–65) for mortality [34, 55–58]. Finally, RV dysfunction is assessed by computerised tomography pulmonary angiography (CTPA), and has been proposed as a possible prognostic tool [1, 2, 59–61]. Recently, right-to-left ventricular dimensional ratio ≥0.9 at CTPA has been suggested to be an independent predictor of early mortality, with a 100% (95% CI 98–100%) negative predictive value for inhospital death [61]. Although it is a promising prognostic test, these findings must be confirmed in other studies.

## 3. Home Treatment and Early Discharge

Worldwide, complete and partial (i.e., early discharge) outpatient treatment is commonly used for patients at low risk of adverse events. Community-acquired pneumonia is a classical example: patients can be treated completely at home, in a clinical ward or, at the opposite site, in an intensive care unit based on the severity of the disease. Among thrombotic disorders, deep vein thrombosis (DVT) is frequently treated completely at home [3]. However, the choice of the best therapeutic setting is not based only on disease-related criteria. Several social circumstances should be concomitantly present: well-maintained living conditions, strong support from family or friends, phone access, and ability to quickly return to hospital if there is deterioration. Moreover, patient should feel well enough to be treated at home [3].

### 3.1. Pulmonary Embolism

Emergency physicians are often reluctant to discharge patients with PE for outpatient treatment due to insufficient data supporting the effectiveness and safety of the outpatient management of PE [62, 63]. Indeed, safe outpatient management of patients with PE may lead to a decrease in unnecessary hospitalizations, reducing the risk of acquired infections and death and to an improvement in health-related quality of life and a reduction in health care costs [4, 5]. Moreover, new oral anticoagulant agents may further simplify PE home therapy avoiding administration of low-molecular-weight heparin (LMWH): many patients are not familiar with subcutaneous injections, and a home-care nurse is often needed.

Several published studies have already provided valuable data supporting a safe home treatment for low-risk PE patients [4, 5]. Most of these promising data are derived from cohorts of PE patients that were prospectively selected for home treatment based on a list of inclusion/exclusion criteria and were recently described in a systematic review [4]. Until 2009, a total of 25 studies on PE outpatient treatment and early discharge have been published [64–88]. From 2009, four additional cohort studies and two randomized clinical trials (RCTs) have been published [89–94]. 

Overall, however, in only 15 manuscripts a complete quality assessment and data extraction were possible [65, 69, 70, 72, 77, 79–82, 85, 87, 89–92]. A total of 1952 patients were analysed in these 15 observational studies. Nine observational studies had a prospective cohort design.

All studies used a classical list of PE-related (e.g., arterial oxygen saturation, clot extension on imaging test), drug-related (e.g., high-risk of major bleeding, creatinine clearance < 30 mL/min), and patient-related (e.g., concomitant medical condition, likelihood of poor compliance) inclusion/exclusion criteria. In the study by Agterof and colleagues a blood test, NT-proBNP level < 500 pg/mL, was used as main criterion for home treatment [91].

In 11 studies patients were discharged from the emergency room within a maximum of 24 h after admission. In the remaining 4 studies, early discharge (1–5 days) was adopted as disposition strategy [70, 72, 77, 79]. An outpatient treatment programme was available in participating centres. This was usually coordinated by a nurse and included the assessment of a physician, if required, an educational session, a daily phone call to each patient, and the availability of a 24 h emergency phone number. Home-care nurses, the patient himself, or a family member administered LMWH. No patient died of pulmonary embolism in the first 7–10 days.

In both RCTs a prognostic CPR, the Uresandi CPR and PESI, was already formally applied to select patients' disposition for the initial treatment of PE [93, 94]. The two studies randomized patients with acute PE to receive LMWH either in the hospital for only 3 days versus entirely in the hospital [93] or entirely out of the hospital (discharged within 24 h) versus at least partly in hospital [94]. The former study was prematurely stopped due to an unexpected high rate of adverse outcomes in included patients at low risk according to Uresandi CPR, even if results were in favour of short hospital stay: in the 132 enrolled patients, overall mortality was 4.2% (95% CI, 0.5–8.9) in the early discharge group and 8.3% (95% CI, 1.1–15) in the standard hospitalization group [93]. In the latter, the so-called OPTE study, 344 patients with PESI I or II class were discharged from the emergency department within 24 hours after randomization or admitted to the hospital and discharged based on the decision of the treating physician [94]. One (0.6%) of 171 outpatients developed recurrent venous thromboembolism within 90 days compared with none of 168 inpatients (95% upper confidence limit (UCL) 2.7%). Only one (0.6%) patient in each treatment group died within 90 days (95% UCL 2.1%), and two (1.2%) of 171 outpatients and no inpatients had major bleeding within 14 days (95% UCL 3.6%). By 90 days, three (1.8%) outpatients but no inpatients had developed major bleeding (95% UCL 4.5%) [94].

The current edition of the ACCP guidelines does not recommend complete home therapy but only suggests “early discharge over standard discharge (e.g., after first 5 days of treatment), in patients with low-risk PE and whose home circumstances are adequate (Grade 2B)”, remarking that “patients who prefer the security of the hospital to the convenience and comfort of home are likely to choose hospitalization over home treatment”. Moreover, the panelists underlie 4 compulsory criteria for out-of-hospital treatment: (1) clinically stable patient with good cardiopulmonary reserve; (2) good social support with ready access to medical care; (3) patient expected to be compliant with followup; (4) absence of severe symptoms or comorbidity.

## 4. New Oral Anticoagulants

Parenteral unfractionated heparin (UFH), LMWH, and fondaparinux are the current options for acute treatment of non-high-risk PE [1–3]. All three options are efficacious but not optimal for home treatment. UFH requires continuous intravenous infusion with frequent laboratory monitoring of activated partial thromboplastin time and dose titration. LMWH and fondaparinux have a more predictable response after administration that allows fixed weight-adjusted doses without the need for laboratory monitoring, but several patients require daily nurse assistance for subcutaneous injections. Moreover, they are retained in patients with renal impairment. Finally, LMWH required platelet monitoring for heparin-induced thrombocytopenia risk [1, 3]. In the last decade, several new oral anticoagulants have been developed and evaluated in RCTs: the direct thrombin inhibitors (e.g., dabigatran) and the factor Xa inhibitors (e.g., rivaroxaban, apixaban, and edoxaban) [95, 96]. The oral route of administration is a major advantage over unfractionated heparin, LMWH, or fondaparinux. Moreover, the possibility to administer these drugs without the need for coagulation test monitoring, thanks to their predictable pharmacokinetic and pharmacodynamic actions and to the limited food-drug and drug-drug interactions, is a major advantage over heparins and, also, over oral vitamin K antagonists (VKAs). Finally, the onset of the anticoagulant activity is rapid, with plasma peak levels of all new anticoagulants that range from 2 to 4 hours after administration. This is undoubtedly a clear advantage in comparison to VKA and may permit a “single-drug” approach for the treatment of acute PE, avoiding initial therapy with parenteral drugs (see Tables 3 and 4).

### 4.1. Direct Thrombin Inhibitors

#### 4.1.1. Dabigatran

Dabigatran etexilate is the prodrug of dabigatran, an univalent, reversible direct thrombin inhibitor that binds exclusively to the active site of thrombin and that dissociates relatively quickly from thrombin [96]. The onset of the anticoagulant activity is rapid, with plasma levels of dabigatran peak at 2 hours. Half-life ranges between 12 and 17 hours. At least 80% of dabigatran is excreted unchanged via the kidneys; therefore, use of dabigatran in patients with severe renal failure, with a creatinine clearance less than 30 mL/min, requires dose reduction and extreme caution [96–100]. Reduced dosages of drug that are substrates for P-glycoprotein (P-gp), such as dabigatran, may be needed when coadministered with strong P-gp inhibitors (e.g., amiodarone, verapamil, and quinidine).

At present, dabigatran is the oral only direct thrombin inhibitors with published phase III trials [101–117]. It is currently licensed in many countries for the prevention of venous thromboembolism (VTE) in patients undergoing hip- and knee-replacement surgery and for the prevention of cardioembolism in patients with nonvalvular atrial fibrillation (AF).

Dabigatran has been evaluated also in two large RCTs focusing on the treatment of acute VTE, the RECOVER and the RECOVER-II studies [118, 119]. In both studies, investigators enrolled patients with acute VTE, DVT, and/or PE, who were initially given parenteral anticoagulation therapy, in a randomized, double-blind, noninferiority trial, aiming to compare dabigatran etexilate, administered at a dose of 150 mg twice daily, with dose-adjusted warfarin (international normalized ratio of 2.0 to 3.0). The choice of the initial parenteral treatment was made in order to ensure the immediate anticoagulant effect and to avoid the high rate of early recurrence, which occurred with the precursor ximelagatran alone [120]. The primary outcome of the studies was the 6-month incidence of recurrent symptomatic, objectively confirmed VTE and related deaths. Safety outcomes included bleeding events, acute coronary syndromes, other adverse events, and results of liver-function tests. In the RECOVER study, 30 of the 1274 (2.4%) dabigatran patients, as compared with 27 of the 1265 (2.1%) warfarin patients, had recurrent VTE [118]. The hazard ratio (HR) with dabigatran was 1.10 (95% CI, 0.65 to 1.84). Major bleeding episodes occurred in 20 (1.6%) dabigatran patients and in 24 (1.9%) warfarin patients (hazard ratio with dabigatran, 0.82; 95% CI, 0.45 to 1.48). The numbers of deaths and abnormal liver-function tests were similar in the two groups. Adverse events leading to discontinuation of the study drug occurred in 9.0% of patients assigned to dabigatran and in 6.8% of patients assigned to warfarin, mainly due to dyspepsia (almost 3% with dabigatran). These results were confirmed in the RECOVER II trial, published only in an abstract form [119]. The only reported side effect of dabigatran in the abstract was dyspepsia, in 3% of patients [119]. 

An increased risk of myocardial infarction has been noted with the use of dabigatran. Two recent meta-analyses have reported an estimated 30% relative risk increase for acute coronary events with the use of dabigatran in patients with VTE and AF [121, 122]. This effect has not been reported with factor Xa inhibitors: it is not clear whether this can be due to an intrinsic property of dabigatran or to a protective effect of the comparator antithrombotic drugs. Before definite data will be provided, it seems wise to prefer other drug options instead of dabigatran in AF patients with known cardiac ischemic disease or at high risk of acute coronary events.

### 4.2. Factor Xa Inhibitors

#### 4.2.1. Rivaroxaban

Rivaroxaban is a potent, selective, and reversible oxazolidinone-based, active-site-directed factor Xa inhibitor. Plasma levels of the drug peak after 3-4 h. Half-life ranges from 5 to 9 h in young individuals and from 11 to 13 h in the elderly. Coadministration of rivaroxaban with food increases the t_max⁡_ from 2.75 to 4 hours, with an increase in the C_max⁡_ and overall exposure of 30–40%. Almost 33% of rivaroxaban is excreted as active metabolites via the kidney [96, 97]. No dose adjustment is required for the elderly, but caution is required for severe renal insufficiency, creatinine clearance 15–30 mL/min [123]. Rivaroxaban is contraindicated in patients with creatinine clearance less than 15 mL/min [123]. Rivaroxaban is also contraindicated in patients receiving concomitant treatment with strong inhibitors of both P-glycoprotein and cytocrome CYP3A4, such as azole compounds and ritonavir. 

Rivaroxaban has been extensively investigated in patients undergoing hip- and knee-replacement surgery, for the treatment of VTE and for the prevention of cardioembolism in patients with nonvalvular AF [124–135]. In particular, rivaroxaban has been specifically evaluated for the treatment of PE in the EINSTEIN-PE trial [136]. Overall, 4832 patients with acute symptomatic PE, with or without DVT, were randomized to rivaroxaban alone (15 mg twice a day for 3 weeks, then 20 mg daily) or standard treatment with therapeutic dose of enoxaparin followed within 48 hours by adjusted-dose VKA for 3, 6, or 12 months. Rivaroxaban results were noninferior to enoxaparin plus VKA in terms of symptomatic recurrent VTE (2.1% versus 1.8%, HR 1.12, 95% CI 0.75–1.68). In particular, the efficacy of rivaroxaban was confirmed also in the early phase, with a similar rate of recurrent events after 3 weeks. Fewer episodes of major bleeding were reported with rivaroxaban compared to standard therapy (1.1% versus 2.2%, HR 0.49, 95% CI 0.31–0.79) [136].

#### 4.2.2. Other Xa Inhibitors

Two other molecules, apixaban and edoxaban, are in a advance stage of development and are currently under investigation for the acute treatment of patients with PE [137–156]. Apixaban is a small molecule that inhibits in a selective and reversible manner the active site of both free and prothrombinase-bound factor Xa [96]. Apixaban has high oral bioavailability (50% to 85%) and a half-life of 12 hours [96, 157, 158]. It is absorbed in the gastrointestinal tract, and its plasma peak is achieved in about 3 h [158]. Food does not interfere with its absorption [96]. Apixaban is metabolized in the liver by cytochrome-dependent and -independent mechanisms, and approximately 25% is cleared through the renal route [96].

Edoxaban tosylate is an oral direct factor Xa inhibitor. The drug is rapidly absorbed, reaching a C_max⁡_ in 1 to 2 hours with a mean plasma half-life of 8 to 10 hours and is mainly eliminated via renal excretion [159]. 

The AMLIFY and the HOKUSAI-VTE studies are the phase III RCTs specifically designed for assessing the benefit-risk profile of apixaban and edoxaban, respectively, for treating VTE. The AMPLIFY study is a phase III trial comparing apixaban 10 mg bid for 7 days then 5 mg, twice daily or to enoxaparin 1 mg/kg bid until INR >2 then warfarin for 6 months for the treatment of patients with acute symptomatic VTE [160]. The HOKUSAI-VTE study is a phase III trial comparing edoxaban tosylate 60 mg once daily or to dose-adjusted warfarin to maintain INR between 2.0 and 3.0, after the initial treatment with parenteral LMWH/unfractionated heparin (minimum 5 days) of patients with acute symptomatic VTE [160].

### 4.3. Other New Drug Option

#### 4.3.1. Idrabiotaparinux

Idraparinux is a long-acting synthetic pentasaccharide highly specific for factor Xa inhibition. It requires only a single weekly subcutaneous injection to obtain its antithrombotic effect [161–163]. 

In the van Gogh trial, 2904 DVT patients and 2215 PE patients received either subcutaneous idraparinux (2.5 mg once weekly) or a heparin followed by an adjusted-dose vitamin K antagonist for either 3 or 6 months [164]. The incidence of recurrence VTE at day 92 was 2.9% in the idraparinux group as compared with 3.0% in the standard-therapy group (odds ratio, 0.98; 95% CI, 0.63 to 1.50) for DVT patients but was 3.4% in the idraparinux group and 1.6% in the standard-therapy group (odds ratio, 2.14; 95% CI, 1.21 to 3.78) for PE patients [164]. In addition to this partially negative results, its prolonged anticoagulant effect raised questions about the potential hazard of bleeding from invasive procedures and the appropriate management of patients with major bleeding.

Therefore, a derivative compound was created. A biotin moiety was added to idraparinux to create idrabiotaparinux, a molecule with the possibility to rapidly reverse its anticoagulant activity by infusing avidin [161, 165, 166].

In the CASSIOPEA study, a noninferiority trial, 3202 PE patients have been randomised to subcutaneous enoxaparin, 1.0 mg/kg twice daily for 5 to 10 days, followed by subcutaneous idrabiotaparinux, 3.0 mg once weekly, and placebo tablets (*n* = 1599) [167] or enoxaparin plus adjusted-dose warfarin (target international normalized ratio 2.0 to 3.0) plus weekly placebo injections after enoxaparin [167]. Primary end point, that is, symptomatic recurrent VTE, occurred in 2.1% of the idrabiotaparinux group and 2.7% of the standard treatment group (relative risk reduction 21%, 95% CI −24 to 49).

## 5. Conclusions

A correct prognostic stratification is the first step for early discharge or a complete home treatment, but a dedicated well-organised 24-h outpatient programme should be provided to each patient, similarly to those already existing for DVT patients all over the world. As acute PE is associated with much higher short-term mortality than acute DVT, outpatient-dedicated programme is crucial to rapidly assess and manage any complication and to correctly investigate PE patient for not missing any possible manifest or occult underlying risk factor, such as cancer.

The availability of new oral anticoagulants may actually simplify the management of PE. These new molecules have remarkable pharmacologic properties (rapid onset of action, short half-life- and predictable anticoagulant effect), which make routine laboratory monitoring of anticoagulation effect unnecessary and may reduce overdosage or bleeding events [91]. Moreover, molecules such as rivaroxaban and apixaban have been tested as “single-drug” for the acute phase, thus making possible the outpatients management of selected patients with PE.

## Figures and Tables

**Table 1:** PESI (pulmonary embolism severity index).

Predictors	Points assigned	Risk class		Points
Age	Age, in yr	I	Very low	≤65
Male sex	+10	II	Low	66–85
Cancer	+30	III	Intermediate	86–105
Heart failure	+10	IV	High	106–125
Chronic lung disease	+10	V	Very high	≥126
Pulse ≥110/min	+20			
Systolic blood pressure <100 mmHg	+30			
Respiratory rate ≥30/min	+20	I-II	Low	≤85
Temperature <36°C	+20	III–V	High	>85
Altered mental status^†^	+60			
Arterial blood oxygen saturation <90%^∣∣^	+20			

^†^Defined as disorientation, lethargy, stupor, or coma.

^∣∣^With and without the administration of supplemental oxygen.

**Table 2:** GENEVA prognostic score.

Predictors	Points assigned	Risk class	Points
Cancer	+2	Low	≤2
Heart failure°	+1	High	>2
Previous DVT	+1		
Systolic blood pressure <100 mmHg	+2		
PaO_2_ < 8 kPa (60 mmHg)^§^	+1		
DVT shown by ultrasound	+1		

°Defined as history of CHF or acute pulmonary edema on the admission chest X-ray.

^§^While breathing room air.

DVT: deep vein thrombosis.

PaO_2_: arterial partial pressure of oxygen.

**Table 3:** Published phase III trials on acute treatment of pulmonary embolism.

Study	Patients	Intervention	Comparison	Primary outcome (recurrent VTE)
RECOVER	Acute symptomatic VTE, DVT, and/or PE, who were initially given parenteral anticoagulation therapy	Dabigatran etexilate (150 mg twice daily)	Dose-adjusted warfarin (INR 2.0 to 3.0)	Dabigatran: 30 (2.4%) Warfarin 27 (2.1%)
RECOVER II	Acute symptomatic VTE, DVT, and/or PE, who were initially given parenteral anticoagulation therapy	Dabigatran etexilate (150 mg twice daily)	Dose-adjusted warfarin (INR 2.0 to 3.0)	Dabigatran: 30 (2.4%) Warfarin 28 (2.2%)
EINSTEIN-PE	Acute symptomatic PE	Rivaroxaban (15 mg bid for the first 3 weeks followed by 20 mg od)	LMWH and dose-adjusted VKA (INR 2.0 to 3.0)	Rivaroxaban: 50 (2.1%) LMWH/VKA: 44 (1.8%)
CASSIOPEA	Symptomatic PE with or without symptomatic DVT, who were initially given parenteral anticoagulation therapy	Idrabiotaparinux 3.0 mg s.c. once weekly	Dose-adjusted warfarin (INR 2.0 to 3.0)	Idrabiotaparinux: 34 (2.1%) Warfarin: 43 (2.7%)

VTE: venous thromboembolism; DVT: deep venous thrombosis; PE: pulmonary embolism; LMWH: low-molecular-weight heparin; VKA: vitamin K antagonist.

**Table 4:** Ongoing phase III trials on acute treatment of pulmonary embolism.

Study	Patients	Intervention	Comparison
AMPLIFY	Acute symptomatic VTE	Apixaban: 10 mg bid for 7 days then 5 mg bid	Enoxaparin and dose-adjusted warfarin (INR 2.0 to 3.0)
HOKUSAI-VTE	Acute symptomatic VTE, who were initially given parenteral anticoagulation therapy	Edoxaban 60 mg once daily	Dose-adjusted warfarin (INR 2.0 to 3.0)

VTE: venous thromboembolism.

## Abbreviations

ACCP: American College of Chest Physicians
AF: Atrial fibrillation
AHA: American Heart Association
BNP: Brain natriuretic peptide
CI: Confidence interval
CPR: Clinical prediction rule
CTPA: Computed tomography pulmonary angiography
DVT: Deep vein thrombosis
ESC: European Society of Cardiology
HR: Hazard ratio
LMWH: Low molecular weight heparin
PE: Pulmonary embolism
PESI: Pulmonary embolism severity index
P-gp: P-glycoprotein
RCT: Randomised controlled trial
RV: Right ventricular
VKA: Vitamin K antagonists
VTE: Venous thromboembolism
UCL: Upper confidence limit
UFH: Unfractionated heparin.
